# Tumor-Targeted Squaraine Dye for Near-Infrared Fluorescence-Guided Photodynamic Therapy

**DOI:** 10.3390/ijms25063428

**Published:** 2024-03-18

**Authors:** Yoonbin Park, Min Ho Park, Hoon Hyun

**Affiliations:** 1 Department of Biomedical Sciences, Chonnam National University Medical School, Hwasun 58128, Republic of Korea; unb1n0213@naver.com; 2 BioMedical Sciences Graduate Program (BMSGP), Chonnam National University, Hwasun 58128, Republic of Korea; 3 Department of Surgery, Chonnam National University Medical School and Hwasun Hospital, Hwasun 58128, Republic of Korea; mhpark@chonnam.ac.kr

**Keywords:** squaraine dyes, reactive oxygen species, photodynamic therapy, near-infrared fluorescence imaging, tumor targeting

## Abstract

Many efforts have been made to develop near-infrared (NIR) fluorescent dyes with high efficiency for the NIR laser-induced phototherapy of cancer. However, the low tumor targetability and high nonspecific tissue uptake of NIR dyes in vivo limit their applications in preclinical cancer imaging and therapy. Among the various NIR dyes, squaraine (SQ) dyes are widely used due to their high molar extinction coefficient, intense fluorescence, and excellent photostability. Previously, benzoindole-derived SQ (BSQ) was prepared by incorporating carboxypentyl benzoindolium end groups into a classical SQ backbone, followed by conjugating with cyclic RGD peptides for tumor-targeted imaging. In this study, we demonstrate that the structure-inherent tumor-targeting BSQ not only shows a high fluorescence quantum yield in serum but also exhibits superior reactive oxygen species (ROS) generation capability under the 671 nm laser irradiation for effective photodynamic therapy (PDT) in vitro and in vivo. Without targeting ligands, the BSQ was preferentially accumulated in tumor tissue 24 h post-injection, which was the optimal timing of the laser irradiation to induce increments of ROS production. Therefore, this work provides a promising strategy for the development of photodynamic therapeutic SQ dyes for targeted cancer therapy.

## 1. Introduction

Photodynamic therapy (PDT) has been extensively applied to treat various types of cancers due to its high activity, low toxicity, excellent spatiotemporal selectivity, and minimally invasive properties [1,2,3]. The photodynamic cancer treatment necessarily must be combined with three key components: light, a photosensitizing agent, and oxygen. Basically, the mechanism of PDT action involves light irradiation of targeted photosensitizers with the right laser wavelengths to produce cytotoxic reactive oxygen species (ROS) specifically in tumors, therefore inducing cancer cell apoptosis [4,5]. Many types of photosensitizing agents have been developed for utilization in the PDT application, including boron dipyrromethene (BODIPY) dyes, cyclic tetrapyrroles (mainly chlorins, porphyrins, and bacteriochlorins), phenothiazinium dyes, and cyanine dye-based photosensitizers [6,7,8]. Since most photosensitizing dyes available commercially exhibit a lack of tumor-targeting specificity, it is very important to develop tumor-targetable photosensitizers used for simultaneous tumor-targeted imaging and efficient photodynamic cancer therapy [9,10].

Among the different types of photosensitizers, squaraine (SQ) dyes have been studied as promising candidates for potentially overcoming several limitations related to conventional photosensitizers. For the light-dependent ROS production, the symmetrical SQ dyes have π-conjugated donor-acceptor-donor structures containing the central acceptor squaryl core edged by the donor aromatic or heterocyclic rings on either side. Previously, Santos et al. reported several heterocyclic SQ dyes varied with the donor moieties of quinoline and benzothiazole side groups. Importantly, the efficiency of the triplet–singlet interconversion of oxygen is significantly dependent on the nature of the terminal heteroaromatic nuclei [11]. Thus, it is very promising to develop SQ-type photosensitizing agents with efficient ROS generation capability for effective PDT in cancer treatment.

Moreover, SQ dyes typically displayed narrow and strong absorption and fluorescence emission bands in the near-infrared (NIR) region with high molar extinction coefficients (~10^5^ M^−1^ cm^−1^), enhanced brightness, and superior photostability [12,13]. Interestingly, the absorption profile of SQ dyes could be varied depending on the substitution of the aromatic rings with polyaromatic or heterocyclic rings. Although the phloroglucinol-based SQ dyes showed sharp absorption peaks in the 550–600 nm region with lower molar extinction coefficients (~10^4^ M^−1^ cm^−1^), the benzothiazole-based SQ dyes exhibited more red-shifted absorption peaks in the 650–700 nm region. Additionally, the absorption maximum of the quinaldine-based SQ dyes is further red-shifted in the 700–750 nm region [14]. Therefore, the intense absorption in the photodynamic window (600–850 nm) makes the benzothiazole- or quinaldine-based SQ dyes suitable for PDT applications.

Several studies have been addressed to overcome two major drawbacks of SQ dyes, such as intrinsic chemical instability and self-aggregation behavior. In particular, the self-aggregation property of SQ dyes in aqueous environments may cause a serious problem during biological applications [15]. Arunkumar et al. developed SQ-based rotaxanes by forming a mechanically interlocked structure to improve the aggregation and photophysical properties of SQ dyes, therefore increasing the generation efficiency of singlet oxygen [16]. Arun et al. reported that the inclusion complex formation of SQ dyes with β-cyclodextrin not only improved the water solubility of SQ dyes but also prevented the self-aggregation behavior of SQ dyes in aqueous solutions [17]. Moreover, this complex formation system can be highly advantageous for the protection of chemically vulnerable squaryl rings, which can easily be attacked by nucleophilic cysteine and glutathione present in the biological system.

Previously, Park et al. developed the benzoindole-derived SQ (BSQ) prepared by incorporating carboxypentyl benzoindolium end groups into a classical SQ backbone, followed by conjugating with cyclic RGD peptides for tumor-targeted fluorescence and photoacoustic imaging [18]. Herein, the BSQ dye is used for structure-inherent tumor targeting without conjugation with tumor-targeting ligands and further applied to photodynamic cancer treatment combined with an NIR laser. To enhance the tumor targetability of BSQ, bovine serum albumin (BSA) was employed as a BSQ delivery carrier for tumor-targeted imaging and efficient PDT. Compared with rotaxane and cyclodextrin in terms of cost, biocompatibility, and tumor targetability, BSA is a better candidate for improving water solubility, photophysical properties, and tumor accumulation of BSQ.

In this study, we demonstrated that BSQ not only displayed a high fluorescence quantum yield in serum rather than saline but also showed excellent ROS production capability under the 671 nm laser irradiation for effective PDT in vitro and in vivo. Without targeting ligands, the BSQ dissolved in serum was preferentially accumulated in tumor tissue 24 h post-injection, which was the optimal timing of the laser irradiation to induce increments of ROS production. Therefore, this work provides a promising strategy for the development of photodynamic therapeutic SQ dyes for targeted cancer therapy.

## 2. Results

### 2.1. Optical Characterization and In Vitro ROS Assay

**Figure 1:**
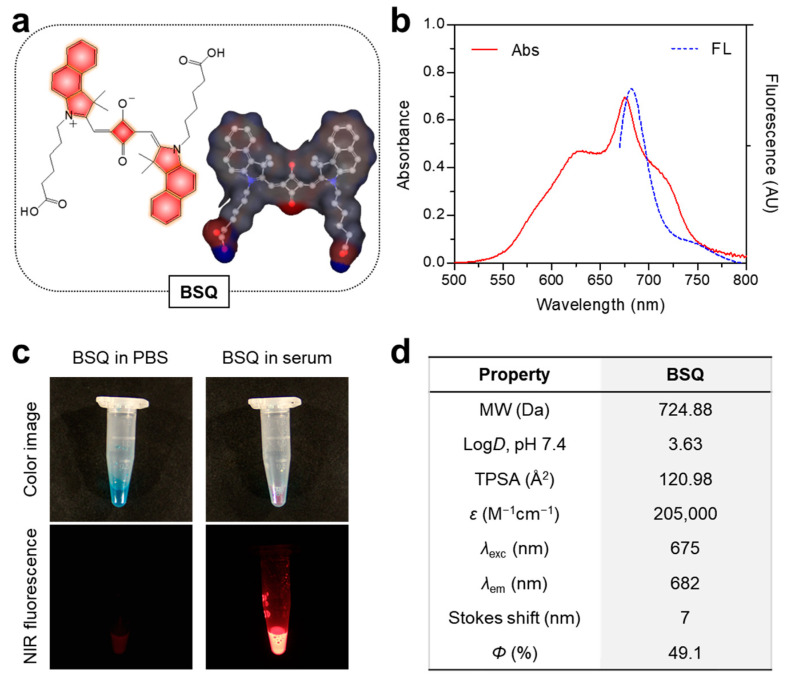
(**a**) Chemical structure and (**b**) absorption and fluorescence emission spectra of BSQ measured in serum. (**c**) Color and NIR fluorescence images of BSQ dissolved in PBS and FBS, respectively. (**d**) Physicochemical and optical properties of BSQ. In silico calculations of the distribution coefficient (log*D* at pH 7.4) and polarity (TPSA) were performed using Marvin and JChem calculator plugins (ChemAxon).

The chemical structure and three-dimensional modeling of BSQ used in this study are displayed in Figure 1a for the prediction of physicochemical properties such as hydrophobicity and polarity. As shown in Figure 1b, the absorption and fluorescence emission spectra of BSQ were measured in fetal bovine serum (FBS) rather than phosphate-buffered saline (PBS, pH 7.4). The peak absorption at 675 nm and maximum fluorescence emission at 682 nm were determined with a small Stokes shift (7 nm), respectively. Interestingly, the absorption spectrum of BSQ in serum shows broad shoulders on both blue and red-shifted aggregates from the main peak. This suggests that the BSQ structure can form both H- and J-aggregates in serum. Importantly, the fluorescence brightness of BSQ exhibited significant differences between PBS and FBS conditions (Figure 1c). Although BSQ is well dispersed in PBS without any precipitation, the fluorescence intensity decreased greatly as compared to FBS, owing to its self-aggregation behavior. This indicates that serum proteins may partly prevent the aggregation property of BSQ, therefore increasing the fluorescence brightness. As summarized in Figure 1d, in silico prediction such as distribution coefficient (log*D* at pH 7.4) and topological polar surface area (TPSA) of BSQ was carried out using ChemAxon’s JChem software (under version 14.12.15.0).

**Figure 2:**
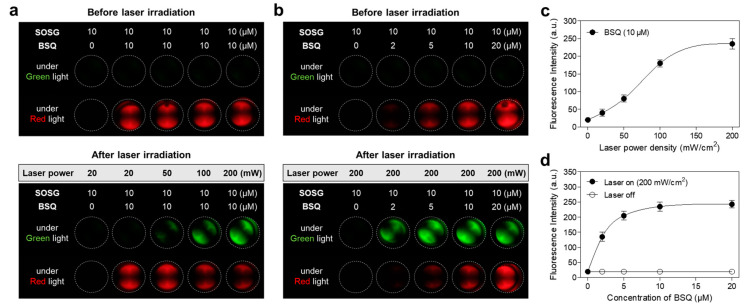
In vitro photodynamic effect of BSQ mixed with SOSG before and after 671 nm laser irradiation for 1 min. ROS generations of BSQ depending on (**a**) the laser power density and (**b**) the BSQ concentrations were analyzed using the 10 μM concentration of SOSG. (**c**) SOSG fluorescence intensity depends on the laser power densities in the range of 20–200 mW/cm^2^ using the 10 μM concentration of BSQ. (**d**) SOSG fluorescence intensity depends on the BSQ concentrations (2–20 μM) under 671 nm laser irradiation at 200 mW/cm^2^ for 1 min.

ROS generation from BSQ was examined using commercially available Singlet Oxygen Sensor Greens reagent (SOSG) as a singlet oxygen sensor to evaluate the photodynamic effect of BSQ. The significant fluorescence signals of SOSG mixed with BSQ were monitored under green light after 1 min laser irradiation. To optimize the power density of the 671 nm laser, the mixtures of SOSG (10 μM) and BSQ (10 μM) were exposed to 671 nm laser irradiation with different power densities (20–200 mW/cm^2^) for 1 min, respectively (Figure 2a). The fluorescence intensities of SOSG correspond to the increased power density of the laser (Figure 2c). Additionally, the efficiency of ROS generation was measured using various concentrations of BSQ (2–20 μM) mixed with SOSG (10 μM) under 671 nm laser irradiation at 200 mW/cm^2^ for 1 min (Figure 2b). The fluorescence signals of SOSG increased up to 5 μM of BSQ and reached a plateau in the concentration range of 10–20 μM (Figure 2d). This result demonstrates that BSQ can effectively generate ROS and act as a photosensitizing agent for photodynamic cancer treatment. For further in vitro studies, the combination of BSQ (10 μM) and laser (200 mW/cm^2^) is the optimal PDT condition.

### 2.2. In Vitro Cytotoxicity and Intracellular ROS Generation

**Figure 3:**
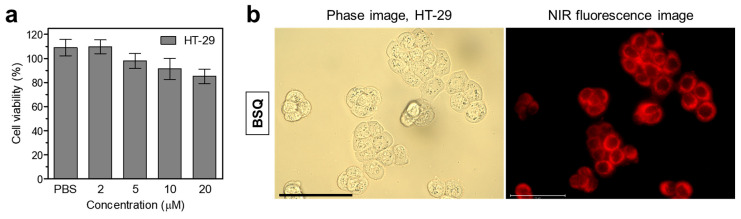
(**a**) Cell viability assay of BSQ using HT-29 cancer cells. Percentage cytotoxicity is determined after 4 h of treatment with various concentrations of BSQ. (**b**) Live-cell binding of BSQ in HT-29 cancer cells. Phase contrast and NIR fluorescence images are obtained after incubation with 10 μM of BSQ for 4 h. Images are representative of n = 3 independent experiments. All fluorescence images had identical exposure times and normalization. Scale bars = 100 μm.

The cell viability and intracellular binding of BSQ were carried out using the 3-(4,5-dimethylthiazol-2-yl)-2,5-diphenyltetrazolium bromide (MTT) assay in HT-29 cancer cells after incubation with BSQ at various concentrations (2–20 μM) for 4 h (Figure 3a). Interestingly, BSQ exhibited no significant cytotoxicity to the HT-29 cancer cells even at the high concentration of 20 μM. This result proves that BSQ has good biocompatibility, which means BSQ should be combined with an NIR laser for effective photodynamic cancer therapy. Moreover, we observed the intracellular binding of BSQ after 4 h of incubation in HT-29 cells. As shown in Figure 3b, BSQ displayed distinct intracellular localization with strong NIR fluorescence signals in the cytoplasm. The high cellular uptake makes BSQ suitable for PDT applications.

**Figure 4:**
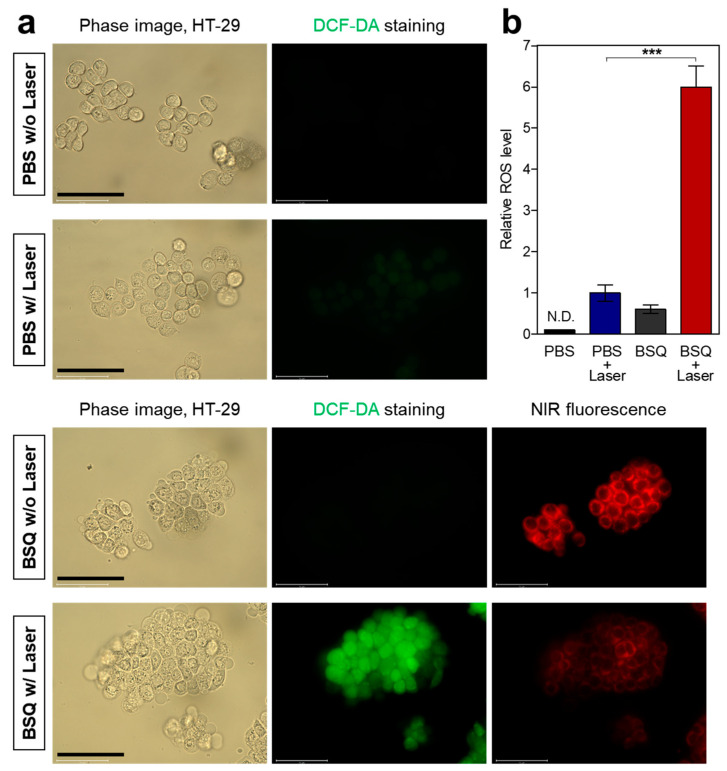
(**a**) Determination of cellular ROS by DCF-DA assay. HT-29 cells were incubated with PBS or 10 μM of BSQ for 4 h and treated with a 671 nm laser at 200 mW/cm^2^ for 5 min. Images are representative of n = 3 independent experiments. All fluorescence images had identical exposure times and normalization. Scale bars = 100 μm. (**b**) Quantification of relative fluorescence intensity after different treatments. Data are expressed as mean ± S.D. (n = 3). *** *p* < 0.001, N.D., not detected.

After confirming the cellular uptake of BSQ, we explored whether BSQ could produce ROS at the cellular level using 2′,7′-dichlorodihydrofluorescein diacetate (DCF-DA) to detect intracellular ROS generation. Cellular ROS production is commonly estimated by nonfluorescent DCF-DA, which permeates the cell membrane and reacts with reactive oxygen to give a DCF form that emits green fluorescence. HT-29 cells were incubated with PBS or 10 μM of BSQ for 4 h and treated with DCF-DA, followed by 671 nm laser irradiation at 200 mW/cm^2^ for 5 min. Importantly, strong green fluorescence signals were detected in the group of BSQ with laser irradiation, which indicates the effective ROS production of BSQ (Figure 4a). As expected, the groups of PBS without laser, PBS with laser, and BSQ without laser irradiation displayed almost no fluorescence, suggesting that the intracellular ROS can only be produced by the combination of BSQ and laser irradiation (Figure 4b). In addition, the NIR fluorescence signals of BSQ in the cells decreased considerably after laser irradiation, indicating that BSQ can be degraded after ROS production.

**Figure 5:**
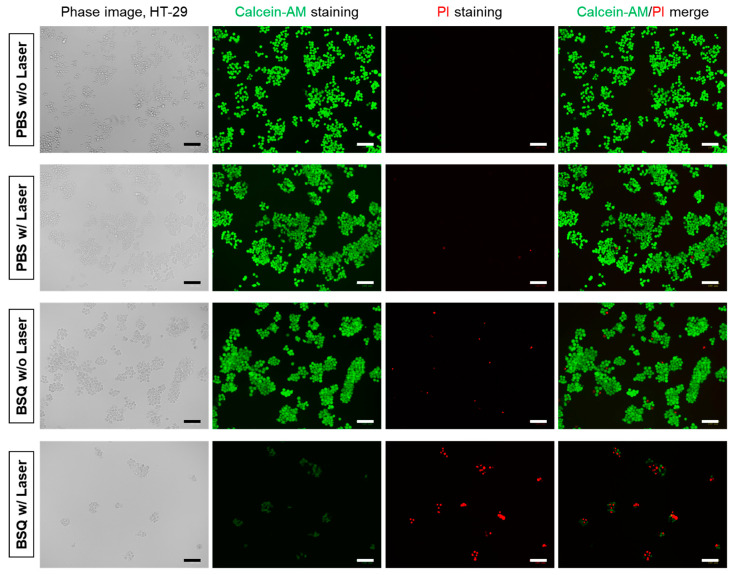
Fluorescence images of HT-29 cells before and after PDT treatment. HT-29 cells were incubated with the 10 µM concentration of BSQ for 4 h and treated with a 671 nm laser at 200 mW/cm^2^ for 5 min. HT-29 cells were then costained with calcein-AM (green for live cells) and propidium iodide (PI; red for dead cells). Images are representative of n = 3 independent experiments. All fluorescence images had identical exposure times and normalization. Scale bars = 100 μm.

To study the in vitro cell phototoxicity before and after laser irradiation, HT-29 cells containing BSQ were exposed to 671 nm laser irradiation with a power density of 200 mW/cm^2^ for 5 min. Compared to the groups of PBS without laser, PBS with laser, and BSQ without laser irradiation, the treatment group of BSQ with laser irradiation exhibited significantly higher amounts of cell death (Figure 5). The green fluorescent cells stained with Calcein-AM decreased apparently, while the red fluorescent cells stained with propidium iodide (PI) increased after laser irradiation. Moreover, large numbers of dead cells were washed out during the step of Calcein-AM/PI double staining, indicating that the combination of BSQ and laser irradiation is highly effective for photodynamic cancer treatment.

### 2.3. Time-Dependent In Vivo Tumor Retention and Photothermal Effect Test

**Figure 6:**
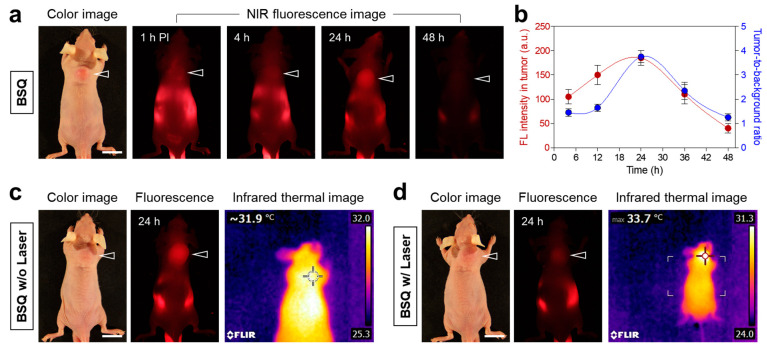
In vivo HT-29 tumor-targeting efficiency of BSQ. (**a**) NIR fluorescence imaging for 48 h after injection of BSQ. (**b**) Time-dependent fluorescence intensity and tumor-to-background ratio at the tumor site targeted by BSQ. NIR fluorescence and thermal images of tumor-bearing mice (**c**) before and (**d**) after laser irradiation (*λ* = 671 nm, 400 mW/cm^2^) for 20 min. The tumor sites are indicated by arrowheads. Scale bars = 1 cm. Images are representative of 3 mice per treatment group. All NIR fluorescence images had identical exposure times and normalization.

To confirm the tumor-targeting ability of BSQ, HT-29 tumor-bearing mice were subjected to a single intravenous injection of BSQ (0.6 mg/kg). It is noticeable that a significant accumulation of BSQ in the tumor site is observed at 24 h post-injection (Figure 6a). According to the fluorescence intensity profile, the BSQ accumulation in the tumor continuously increased up to 24 h after injection and decreased gradually within 48 h after injection (Figure 6b). Based on this result, the tumor-targeted imaging and photodynamic treatment using the BSQ and NIR laser can be carried out optimally at 24 h post-injection. Additionally, we investigated whether BSQ could generate photothermal energy after the 671 nm laser irradiation for the synergistic effect of PDT and photothermal therapy (PTT). An infrared thermal imager repeatedly monitored the tumor temperatures before and after laser irradiation. Unfortunately, the BSQ accumulated in the tumor site displayed no photothermal effect after the 671 nm laser irradiation at 400 mW/cm^2^ for 20 min (Figure 6c,d). This indicates that BSQ can only be utilized for photodynamic cancer therapy rather than PTT. Interestingly, the fluorescence signal of BSQ accumulated in the tumor area was drastically reduced after the 671 nm laser irradiation (Figure 6d). This suggests that the BSQ structure can be largely photodegraded during exposure to concentrated NIR light. Hence, it is necessary to improve the tumor targetability of photosensitizing agents for efficient PDT applications.

### 2.4. In Vivo Photodynamic Therapeutic Efficacy

**Figure 7:**
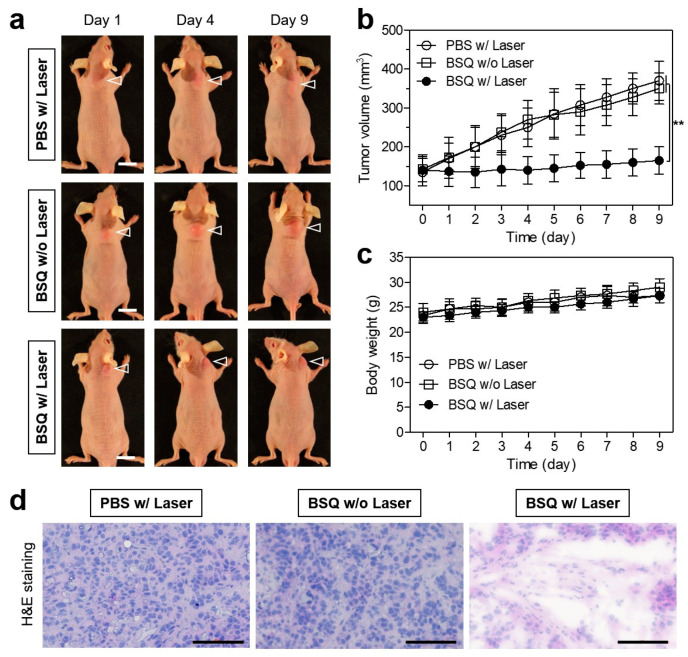
In vivo NIR phototherapeutic efficacy of BSQ. (**a**) Representative photos of changes in tumor size in HT-29 tumor-bearing mice for 9 days after different treatments. The laser groups were treated with 24 h post-injections of PBS or BSQ, followed by 671 nm laser irradiation (400 mW/cm^2^) for 20 min. The tumor sites are indicated by arrowheads. Scale bars = 1 cm. (**b**) Tumor growth rates and (**c**) body weights of each treatment group were monitored for 9 days. Data are expressed as mean ± S.D. (n = 3). ** *p* < 0.01. (**d**) Tumor sections stained with H&E harvested from each group at day 9 after different treatments. Scale bars = 100 μm.

After confirming the markedly increased tumor accumulation of BSQ at 24 h after injection, the efficacy of photodynamic tumor therapy under the 671 nm laser irradiation in the HT-29 cells xenografted nude mouse model was investigated by monitoring the tumor growth in each treatment group for 9 days (Figure 7a). BSQ or PBS alone were injected intravenously into the tumor-bearing mice 24 h before laser irradiation. Subsequently, the tumors were irradiated with the 671 nm NIR laser at 400 mW/cm^2^ for 20 min. In control groups, which were treated with PBS with laser irradiation and BSQ without laser irradiation, tumors grew continuously, and the tumor volumes between the two groups showed no significant difference (Figure 7b). The PBS treatment group exhibited no antitumor effect by only the laser irradiation, whereas the mice treated with BSQ and laser irradiation revealed tumor growth inhibition during the treatment. This result demonstrates that only the combination of BSQ and laser irradiation can induce a significant PDT effect. All treatment groups showed no obvious loss of body weight for 9 days of monitoring (Figure 7c). This indicates that the BSQ-induced PDT system is safe and effective for future clinical applications. Furthermore, histological examination of the tumors harvested at day 9 in each group was conducted by hematoxylin and eosin (H&E) staining (Figure 7d). The tumor sections treated with BSQ and laser irradiation displayed significant features of apoptotic cell death with a reduced cell number and shrunken nuclei, whereas typical morphological features of cell proliferation were observed in the tumor tissues treated with PBS with laser irradiation and BSQ without laser irradiation, respectively. This suggests that the BSQ combined with laser irradiation can be effectively used as a photosensitizing agent for the suppression of tumor growth.

## 3. Discussion

Previously, the carboxylated form of BSQ was designed and synthesized for active tumor targeting after conjugation with a cyclic RGD peptide, which specifically binds integrin α_v_β_3_ overexpressed in various cancer and angiogenic cells. Although the BSQ itself is well dispersed in PBS without precipitation, as shown in Figure 1c, it displays poor tumor accumulation in M21 tumor-bearing mice, compared with that of the cyclic RGD-conjugated BSQ, owing to the lack of tumor-targeting ligands [18]. One of the major drawbacks to the use of SQ dyes in biomedical applications is the aggregation behavior of SQ dyes in aqueous environments, resulting in the loss of their photophysical properties. Based on previous studies, this problem can be addressed using carrier systems, which include serum albumins (human and bovine) and β-cyclodextrin, for improving the bioavailability and photostability of SQ dyes [14]. The albumin-SQ complex alleviated the aggregation-induced quenching in aqueous solutions, allowing the SQ dyes to be brightly fluorescent in physiological conditions [19,20]. To clarify the site-selective binding of SQ into the BSA binding domain, Gao et al. demonstrated that the SQ molecule was found to bind in both domain II and domain III, which has the hydrophobic binding pocket with largely reduced solvent-accessible surface area [21]. Thus, the binding of SQ molecules to BSA is very stable owing to the strong hydrophobic interaction.

To enhance the tumor targetability of BSQ without the conjugation of targeting ligands, a saline solution containing 5% wt./v BSA for intravenous injection was alternatively used in this study as a promising carrier for tumor-targeted BSQ delivery. It is well-known that albumin can accumulate selectively into the tumor interstitium, which is the enhanced permeation and retention (EPR) effect. In addition, the EPR effect of albumin can be explained by receptor-mediated albumin uptake pathways that are involved with albumin-binding membrane proteins such as glycoprotein 18 (gp18), gp30, gp60 (albondin), cubilin, megalin, secreted protein acidic and rich in cysteine (SPARC), and the neonatal Fc receptor for IgG (FcRn) [22]. Consequently, the albumin can serve as a tumor-targeting carrier for enhanced tumor accumulation of BSQ, enabling tumor-targeted imaging and effective PDT.

Typically, the mechanism of PDT action using SQ dyes involves two main processes: type I, generation of radical species (such as hydroxyl radicals) via electron transfer, and type II, generation of singlet oxygen (^1^O_2_) via electron spin change [23]. More importantly, type I PDT is independent of O_2_, which has great potential for efficient PDT performance of hypoxic tumors, unlike type II PDT. Although it is controversial to follow the type I or type II mechanism, both type I and type II reactions can damage cellular constituents and eventually cause cell death [24,25]. Taking the above points into consideration, BSQ may be explained by both type I and II mechanisms because BSQ generates the singlet oxygen determined by the SOSG assay and produces the hydroxyl radicals confirmed by the DCF-DA assay.

According to Kasha’s exciton theory, the cofacially stacked H-aggregation of dyes appears to have a rapid internal conversion of the higher excited state into the lower energy exciton state, which quenches the fluorescence and thus may facilitate heat generation [26]. Although BSQ used in this study revealed only PDT capability rather than photothermal effect, we suggest that BSQ can be utilized for the PTT application after incorporation of BSQ within various nanocomposites to form stable H-dimeric SQ nanostructures, which display intense NIR-II (1000–1700 nm) absorption, highly quenched fluorescence, excellent photostability and photothermal conversion efficiency [27]. This strategy provides an advantage over the PDT for hypoxic tumors, which weakens type II PDT performance. Therefore, we highlight the future perspective of BSQ for synergistic PDT/PTT combination therapy, which may be more effective in treating cancers.

In summary, we demonstrated that the BSQ dissolved in serum can effectively serve as a tumor-targeted fluorescence imaging agent in the NIR window and as a ROS-generating PDT agent without photothermal effect under the 671 nm laser irradiation. This tumor-targeted PDT agent holds great potential for simultaneous tumor diagnosis and phototherapy in the future. Furthermore, this study provides a promising strategy for the development of photodynamic therapeutic SQ dyes for targeted cancer therapy.

## 4. Materials and Methods

### 4.1. Optical and Physicochemical Property Analyses

BSQ was kindly provided by Park’s lab (Biological Resource Center, Korea Research Institute of Bioscience and Biotechnology, Jeongeup, Republic of Korea) [18]. All optical measurements were performed in PBS at pH 7.4 (Sigma–Aldrich, St. Louis, MO, USA) or FBS (Welgene, Daegu, Republic of Korea). The absorption spectrum of BSQ was measured by a fiber optic FLAME spectrometer (Ocean Optics, Dunedin, FL, USA). The molar extinction coefficient (*ε*) of BSQ was calculated using the Beer-Lambert equation. The fluorescence emission spectrum of BSQ was recorded using a SPARK^®^ 10M microplate reader (Tecan, Männedorf, Switzerland) at an excitation wavelength of 620 nm and emission wavelengths ranging from 670 to 800 nm. To determine the fluorescence quantum yield (*Φ*), oxazine 725 in ethylene glycol (*Φ* = 19%) was used as calibration standards under the conditions of matched absorbance at 655 nm [28]. In silico predictions of the distribution coefficient (log*D* at pH 7.4) and polarity (TPSA) were performed using Marvin and JChem calculator plugins (ChemAxon, Budapest, Hungary).

### 4.2. In Vitro Live-Cell Imaging

The human colorectal adenocarcinoma cell line (HT-29) was obtained from the American Type Culture Collection (ATCC; Manassas, VA, USA). HT-29 cells were cultured in Roswell Park Memorial Institute (RPMI) 1640 medium (Gibco BRL, Paisley, UK) containing FBS, penicillin, streptomycin, and amphotericin B (Welgene) on a culture plate. The cultured cells were stored in a humidified incubator set to 5% CO_2_ at 37 °C. Fluorescence microscopic imaging was performed using a 4-filter set of the Nikon Eclipse Ti-U inverted microscope system (Nikon, Seoul, Republic of Korea). Image acquisition and analysis were performed using the NIS-Elements Basic Research software (Nikon) https://www.microscope.healthcare.nikon.com/products/software/nis-elements/nis-elements-basic-research (accessed on 11 March 2024). All fluorescence images had identical exposure time and normalization.

### 4.3. In Vitro Cytotoxicity Assay

When the HT-29 cells reached a confluence of approximately 50%, cell toxicity, and proliferation were evaluated using an MTT (Sigma–Aldrich) assay. HT-29 cells were seeded onto 96-well plates (1 × 10^4^ cells per well). To evaluate the cytotoxicity depending on the BSQ concentration, the cancer cells were treated with BSQ (2, 5, 10, and 20 μM) for 4 h and cultured for 24 h after treatment. At each time point, the incubation cell medium was replaced with 100 μL of fresh medium, and 10 μL of the MTT solution was directly added to each 100 μL well. Subsequently, the plates were then incubated for 4 h at 37 °C in a humidified 5% CO_2_ incubator. Finally, the plates were placed in a microplate reader (SPARK^®^ 10M, Tecan) to measure the absorption intensity at 570 nm. Cell viability was calculated using the following formula: cell viability (%) = (*A*_sample_ − *A*_blank_)/(*A*_control_ − *A*_blank_) × 100, where *A* is the average absorbance.

### 4.4. In Vitro Cellular ROS Assay and Photodynamic Cytotoxicity

HT-29 cancer cells were incubated with the 10 µM concentration of BSQ for 4 h. Subsequently, the cells were washed with PBS and treated with a 100 µM concentration of DCF-DA (Thermo Fisher Scientific, Waltham, MA, USA) for 30 min at 37 °C. After laser irradiation (λ = 671 nm, 200 mW/cm^2^) for 5 min, the cells were repeatedly washed with PBS and then observed under the fluorescence microscope (Nikon). For the evaluation of the PDT effect, the cells were allowed to incubate for another 3 h and then costained with calcein-AM and PI (Thermo Fisher Scientific) for 30 min. After washing twice with PBS, the stained cells were observed under the fluorescent microscope.

### 4.5. HT-29 Xenograft Mouse Model

Animal studies were performed in accordance with the guidelines approved by the Chonnam National University Animal Research Committee (CNU IACUC-H-2023-57). Adult (6-week-old, ≈25 g) male athymic nude mice were purchased from OrientBio (Gwangju, Republic of Korea). HT-29 cancer cells were cultured and suspended in 100 μL of PBS before being subcutaneously inoculated in the right flank of each mouse (1 × 10^6^ cells per mouse). When tumor sizes reached about 1 cm in diameter between 8 to 10 days post-inoculation, BSQ dissolved in saline containing 5% wt./v BSA was administered intravenously. Animals were euthanized for in vivo NIR fluorescence imaging within a designated period of time.

### 4.6. In Vivo Time-Dependent Tumor Imaging

In vivo NIR fluorescence imaging was performed using a FOBI imaging system (NeoScience, Deajeon, Republic of Korea). Mice (3 mice per treatment group) were imaged for 48 h after injection to confirm the time-dependent tumor accumulation of BSQ. The fluorescence intensity of the tumor site was analyzed using ImageJ software (National Institutes of Health, Bethesda, MD, USA, https://imagej.net/ij/). Temperature changes at the tumor site were monitored using a thermal imager (FLIR Systems, Wilsonville, OR, USA).

### 4.7. In Vivo Photodynamic Therapeutic Efficacy

BSQ or PBS was intravenously injected into the HT-29 tumor-bearing mice (3 mice per treatment group), and the mice were anesthetized after 24 h. The tumors were treated with a laser (*λ* = 671 nm, 400 mW/cm^2^) for 20 min. Tumors were harvested from each treatment group at day 9 after irradiation for subsequent analysis of histological samples stained with H&E. To assess the in vivo antitumor effect, the macroscopic tumor growth of each group was observed for 9 days. The tumor volume (V) was measured by the following formula: V = 0.5 × longest diameter × (shortest diameter)^2^.

### 4.8. Histological Analysis

Resected tumors were preserved for H&E staining and microscopic observation. The tumors were fixed in 4% paraformaldehyde and flash-frozen in an optimal cutting temperature (OCT) compound using liquid nitrogen. Frozen samples were cryosectioned (10 µm thick), stained with H&E, and observed using a microscope. Histological analysis was performed on the Nikon Eclipse Ti-U inverted microscope system. Image acquisition and analysis were performed using the NIS-Elements Basic Research software, https://www.microscope.healthcare.nikon.com/products/software/nis-elements/nis-elements-basic-research.

### 4.9. Statistical Analysis

A one-way analysis of variance (ANOVA) was performed, followed by Tukey’s multiple comparison test. The results were represented as mean ± standard deviation (S.D.). A value of *p* < 0.05 was considered statistically significant. Curve fitting was performed using the Prism software version 5.01 (GraphPad, San Diego, CA, USA).

## Data Availability

Data are contained within the article.
